# Role of Immune Checkpoint Inhibitors in Gastrointestinal Malignancies

**DOI:** 10.3390/jcm9082533

**Published:** 2020-08-06

**Authors:** Anita Mazloom, Nima Ghalehsari, Victor Gazivoda, Neil Nimkar, Sonal Paul, Peter Gregos, Janice Rateshwar, Uqba Khan

**Affiliations:** 1 Department of Medicine, NewYork-Presbyterian Brooklyn Methodist Hospital—Weill Cornell Medicine, Brooklyn, NY 11215, USA; anm9366@nyp.org (A.M.); nig9073@nyp.org (N.G.); nsn9004@nyp.org (N.N.); sop9033@nyp.org (S.P.); psg9004@nyp.org (P.G.); janice.rateshwar@jerichoapps.org (J.R.); 2 Department of Surgery, Maimonides Medical Center, Brooklyn, NY 11219, USA; vgazivoda@maimonidesmed.org

**Keywords:** Immune Checkpoint Inhibitors (ICIs), Microsatellite Instability-High (MSI-H), Tumor Mutational Burden (TMB), Combined Positive Score (CPS), immunotherapy, gastrointestinal malignancies

## Abstract

Immune checkpoint inhibitors (ICIs) have revolutionized the treatment of several solid and hematological malignancies. ICIs are not only able to produce long and durable responses, but also very well tolerated by patients. There are several approved indications of use of ICIs in treatment of metastatic gastrointestinal malignancies including gastric, esophageal, colorectal and hepatocellular carcinoma. In addition, ICIs can be used in microsatellite instability-high (MSI-H) and high tumor mutational burden (TMB) tumors in chemotherapy-resistant setting. Despite having good efficacy and superior safety profile, ICIs are clinically active in small subset of patients, therefore, there is a huge unmet need to enhance their efficacy and discover new predictive biomarkers. There are several ongoing clinical trials that are exploring the role of ICIs in various gastrointestinal cancers either as single agent or in combination with chemotherapy, radiation therapy, targeted agents or other immunotherapeutic agents. In this review, we discuss the published and ongoing trials for ICIs in gastrointestinal malignancies, including esophageal, gastric cancer, pancreatic, hepatocellular, biliary tract, colorectal and anal cancers. Specifically, we focus on the use of ICIs in each line of therapy and discuss the future directions of these agents in each type of gastrointestinal cancer.

## 1. Introduction

Immunotherapy has been the cornerstone of success in the treatment of several malignancies in the modern era [1]. It has revolutionized the treatment of both solid as well hematologic malignancies. There are several forms of immunotherapies currently being used in clinical practice both as standard of care as well as in the clinical trial setting. Some of the common forms of immunotherapy include ICIs, chimeric antigen receptors, tumor infiltrating lymphocytes, vaccines, oncolytic viruses and therapies directed at natural killer cells and macrophages [2,3]. By far the most important and well-studied immunotherapeutic agents are the ICIs [4]. There are several forms of ICIs targeting at several checkpoint proteins or receptors including programmed cell death 1 (PD-1), PD-1 ligand (PD-L1), cytotoxic T-lymphocyte-associated protein 4 (CTLA-4), B and T cell lymphocyte attenuator (BTLA), V-domain Ig suppressor of T cell activation (VISTA), lymphocyte activation gene 3 (LAG3) and T cell immunoglobulin and mucin domain 3 (TIM-3) [5,6,7,8,9]. ICIs, specifically PD-1, PDL-1 and CTLA-4 inhibitors have been approved for the treatment of a variety of solid tumors, initially beginning with melanoma in 2011. Both PD-1 and CTLA-4 are negative costimulatory molecules that when inhibited enhance T cell activation and the eventual killing of tumor cells [10]. To date, the approved immune checkpoint inhibitors for gastrointestinal malignancies target PD-1/PDL-1 and CTLA-4. There are several biomarkers used in predicting the response to treatment with ICIs [11]. The most important biomarkers that should be checked routinely in clinical practice includes PDL-1, microsatellite instability (MSI) and tumor mutational burden (TMB) [12].

Gastrointestinal malignancies involve several types of malignancies, each of which are treated differently. They are responsible for a large number of cancer related deaths and treatment options are limited especially in those tumors that are metastatic or not amenable to resection. Immunotherapy offers a promising avenue in management of many gastrointestinal malignancies specifically in treatment of gastroesophageal cancers and hepatocellular carcinoma [13]. In addition, ICIs can be used in patients with chemotherapy-resistant tumors through tissue agnostic approval for MSI-H and high mutational burden tumors [14]. ICIs have shown that they are not only efficacious but have superior safety profile as well [15]. Most of the ICIs are well tolerated, however, they have distinct side effects compared to traditional cytotoxic chemotherapies [16,17]. These side effects are termed as immune related adverse events (irAEs). The irAEs can involve virtually any organ of the body including skin, lung, gastrointestinal system, endocrine system and neurological system [18]. Patients should be monitored closely during immunotherapy for development of these irAEs. Usually, the ICIs are stopped or held if irAEs develop and are treated according to grade of toxicity. Immunosuppression, typically in the form of steroids, is usually the backbone of treatment of severe irAEs [19]. Table 1 provides the summary of approved ICIs in various gastrointestinal malignancies. Below, we review the role of checkpoint inhibitors in gastrointestinal malignancies and discuss the future clinical trials in each cancer type. (see Table 1)

## 2. Esophageal & Gastric Cancer

There are approximately 44,000 new cases of esophageal and gastric cancer per year in the United States [20]. Despite the approval of several new systemic therapies, esophageal and gastric cancers remain among the most lethal malignancies in gastrointestinal tract [21]. There are several options available for treatment of advanced or metastatic disease depending upon the line of therapy, the type of histology (squamous versus adenocarcinoma) and the presence of biomarkers (human epidermal growth factor receptor 2 (HER2), MSI and PD-L1. Immunotherapy in the form of checkpoint inhibitor has been approved in different lines of therapy as well.

## 3. Role of ICIs in First Line Setting

The standard of care first line therapy for esophageal and gastric cancers involves cytotoxic chemotherapy with the addition of anti-HER2 targeted therapy in cancers whose cells overexpress the receptor tyrosine kinase HER2. The preferred first-line regimen for unresectable locally advanced, recurrent or metastatic esophageal and gastric cancers is platinum-based therapy with a fluoropyrimidine backbone [22]. Two-drug cytotoxic regimens are generally preferred in this context owing to a decreased risk of toxicity. The role of checkpoint inhibitors has been evaluated in several trials; however, no approval has yet been granted for treatment in the first line setting. Below is a summary of the important clinical trials investigating the role of checkpoint inhibitors in the first line setting.

One of the major trials in first line setting was KEYNOTE-062, which was a phase III randomized clinical trial of 763 patients with advanced gastric or gastroesophageal junction cancer who were randomly assigned to either pembrolizumab at 200 mg every 3 weeks for up to 2 years, placebo plus chemotherapy or pembrolizumab plus chemotherapy (cisplatin and fluorouracil or capecitabine). All eligible patients had PD-L1 combined positive score (CPS) of at least 1. 37%. Of the study participants who had PD-L1 CPS of ≥ 10, 69% of patients had gastric cancer while 30% had GEJ cancer. The primary end point of the study was overall survival. The study demonstrated that overall survival for patients in the pembrolizumab arm was non-inferior to those receiving standard chemotherapy for patients whose tumors had a CPS ≥ 1. Overall, survival (OS) was superior to chemotherapy in the subset of patients receiving pembrolizumab whose tumors had a CPS score ≥ 10. Patient who had received pembrolizumab had median overall survival of 10.6 months compared with 11.1 months for those who received chemotherapy only. However, the pembrolizumab plus chemotherapy arm did not show superior overall survival or progression free survival (PFS) in patients with CPS ≥ 1 [23]. This study showed that single agent pembrolizumab has activity in first line setting, however, it lacks clinically meaningful activity when compared to chemotherapy in patients with CPS ≥ 1.

Another key trial that was performed in the first line setting was the phase III, JAVELIN Gastric 100 trial that compared maintenance therapy with the PD-L1 inhibitor avelumab to the continuation of first line chemotherapy. Patients whose tumors did not progress after 12 weeks of first line chemotherapy (oxaliplatin/fluoropyrimidine induction treatment) were randomly assigned to avelumab 10 mg/kg every 2 weeks and then switched to maintenance or continued on chemotherapy. The primary endpoint was OS post induction therapy in all randomized patients. A total of 499 patients were randomized in this study. The median OS post induction was 10.4 months in avelumab arm compared to 10.9 months in chemotherapy arm (95% CI 9.6–12.4), hazard ratio (HR) 0.91 (95% CI 0.74–1.11 *p* = 0.1779). The study failed to meet the primary objective as no overall survival benefit was observed in either the randomized or PD-L1 positive populations [24]. This study demonstrates no role of switch maintenance of avelumab in first line setting.

In the light of above data, chemotherapy remains the preferred standard of care treatment in first line setting.

## 4. Role of ICIs in Second Line Setting

There are several options available for systemic treatment in second line setting including cytotoxic chemotherapy and targeted therapy like the vascular endothelial growth factor (VEGF) inhibitor, ramucirumab. Similarly, checkpoint inhibitors have been evaluated for use in the second line setting.

The phase III KEYNOTE-061 trial investigated the use of pembrolizumab in 592 patients with advanced gastric or GEJ adenocarcinoma. Eligible patient had CPS ≥ 1 [25]. In this study, patients who had progression of disease after first-line treatment with platinum and fluoropyrimidine doublet therapy either received paclitaxel or pembrolizumab. Notably, patients with squamous cell or undifferentiated gastric cancer, as well as patients with prior immunotherapy were excluded from this study. In this study, pembrolizumab did not significantly prolong overall survival (median 9.1 versus 8.3 months). However, a subgroup analysis demonstrated a significant benefit for use of pembrolizumab over a taxane in patients with a deficient mismatch repair (dMMR) GEJ or gastric cancer. The FDA has approved pembrolizumab and nivolumab in any MSI-H patients with chemotherapy refractory disease. Therefore, pembrolizumab or nivolumab can be used in second line setting for MSI-H gastric or esophageal tumors. Due to this indication, it is imperative to check MSI in all patients.

Another major clinical trial was KEYNOTE-181 phase III trial. In this trial 628 patients with advanced or metastatic esophageal squamous cell carcinoma (SCC) or Siewert type I adenocarcinoma that had progressed after first-line chemotherapy were randomized to either pembrolizumab or the investigator’s choice of standard chemotherapy with paclitaxel, docetaxel or irinotecan [26]. The three co-primary endpoints were OS in the intent-to-treat population, the squamous cell carcinoma subgroup and the subgroup with a CPS ≥ 10. There were 35% of the study population who had CPS ≥ 10. The study did not show OS benefit in intent to treat population. However, this study demonstrated that pembrolizumab significantly improved the median OS (9.3 vs. 6.7 months) in patients whose tumor had a PD-L1 CPS > 10. In July 2019, mainly based on the data from this trial, the FDA approved pembrolizumab in patients with recurrent locally advanced or metastatic SCC of the esophagus who progressed after one or more lines of chemotherapy.

Nivolumab has also been studied in second line setting in advanced SCC of esophagus. ATTRACTION-3 was phase III randomized multicenter clinical trial that randomly assigned 419 patients to either nivolumab or investigator’s choice of chemotherapy (paclitaxel or docetaxel). The primary endpoint was overall survival. The study demonstrated an improved overall survival in patients with previously treated esophageal SCC who received nivolumab versus chemotherapy, irrespective of PD-L1 expression. The median OS was 10·9 months compared to 8.4 months in chemotherapy arm. (HR 0·77, 95% CI 0·62–0·96; *p* = 0·019) [27,28]. Based on these results, FDA approved nivolumab for patients with unresectable advanced, recurrent or metastatic esophageal squamous cell carcinoma after progression on fluoropyrimidine and platinum-based chemotherapy.

## 5. Role of ICIs in Third Line Setting

Pembrolizumab has been approved in third line setting for gastric or gastroesophageal adenocarcinoma. The approval was based on KEYNOTE-059 trial. This was a phase II, single-arm, multicohort study. The primary endpoint was response rate. 259 patients were enrolled into this study, the objective response rate was 11.6% in all patients while it was 15.5% in PD-L1 positive patients. FDA approved pembrolizumab for PD-L1 expressing gastric and gastroesophageal adenocarcinomas after progression on or after two or more prior systemic therapies, including fluoropyrimidine- and platinum-containing chemotherapy [29].

## 6. Ongoing Trials

There are several ongoing clinical trials of ICIs in gastric and esophageal cancers that incorporate immunotherapy. Check-Mate 649 (NCT 02872116) is an ongoing randomized phase III study investigating the use of immunotherapy in previously untreated advanced or metastatic gastric or GEJ cancer. In this study, nivolumab alone—or nivolumab plus ipilimumab in combination with systemic chemotherapy—is being compared to systemic chemotherapy alone in patients who have not received neoadjuvant or adjuvant treatment within the last six months [30]. The primary endpoint is OS in patients with PD-L1 (≥1%) tumors with secondary endpoints including OS in all patients, PFS and time to symptom deterioration in all patients and in those with PD-L1 positive tumors and safety. In addition, there several combination trials of ICIs with other targeted therapies including tyrosine kinase inhibitors and VEGF inhibitors are currently enrolling patients. There are several trials that are combining ICIs with other agents especially immuno-modulating drugs along with radiation therapy to enhance the efficacy of ICIs in gastroesophageal tumors. There are few trials looking at the role of oncolytic virus in combination with ICIs to enhance their efficacy [31]. The role of checkpoint inhibitors in first line setting is being investigated in KEYNOTE-811 (NCT03615326), which is an ongoing randomized, double-blinded phase III trial comparing standard of care chemotherapy (SOC) in combination with trastuzumab versus SOC chemotherapy in combination with pembrolizumab plus trastuzumab in HER-2 positive advanced gastric and gastroesophageal cancer. SOC is defined as Cisplatin on Day 1 and 5-FU on Day 1–5 of each 3-week cycle and [32]. The results of several ongoing trials will likely help expand the role of immunotherapy in management of gastroesophageal tumors.

## 7. Pancreatic Adenocarcinoma

Pancreatic cancer is one of the most aggressive cancer of gastrointestinal system. Globally, pancreatic cancer is the 7th leading cause of cancer deaths [33]. Systemic chemotherapy remains the mainstay of treatment for locally advanced and metastatic pancreatic adenocarcinoma. There are several options available for systemic treatment including FOLFIRINOX and gemcitabine with nab–paclitaxel combination. Despite availability of several cytotoxic chemotherapy options, the median OS of these patients remains dismal. Currently, there is no approved ICIs in pancreatic adenocarcinoma. ICIs can only be used if the tumor is MSI-H or have high tumor mutational burden. However, the patients who harbor MSI-H tumors are a small subset population, counting for less than 2% of all pancreatic adenocarcinoma [34].

The use of immunotherapy in pancreatic adenocarcinoma is limited. It is speculated that the lack of immunogenicity of the tumor limits the use of immunotherapy, which is likely due to the lack of effector T cell activation in tumor cells. There are several trials that have looked at the utility of ICIs in pancreatic adenocarcinoma [35]. Ipilimumab has been investigated as a single agent in phase II clinical trial which showed no evidence of overall survival benefit [36]. Although immunotherapy monotherapy has not shown significant overall survival benefit, the role of dual checkpoint inhibition was also investigated. In a phase II trial that randomized patients to durvalumab with or without tremelimumab in previously treated metastatic pancreatic adenocarcinoma, objective response rate was 3.1% for patients receiving combination therapy and 0% for patients receiving monotherapy. Similarly, median overall survival was 3.6 and 3.1 months in durvalumab monotherapy or durvalumab plus tremelimumab, respectively, after initial therapy with 5-FU or gemcitabine-based therapy [37].

The combination of chemotherapy with immunotherapy has shown some clinical benefit. In a phase I trial of gemcitabine with tremelimumab, two out of twenty-eight patient had partial response and seven patients had stable disease [38]. Similarly, gemcitabine with ipilimumab resulted in partial response in two out of sixteen patients, while five patients had stable disease [39]. In a phase II trial, treatment naïve metastatic pancreatic cancer patients treated with gemcitabine plus nab-paclitaxel, durvalumab, and tremelimumab had a disease control rate of 100% [40]. ICIs have been studied in combination with other agents as well. Combination of CXC chemokine receptor 4 (CXCR4) blocker with PDL-1 and chemotherapy was studied in COMBAT/KEYNOTE-202 (NCT 02826486) trial. In pancreatic adenocarcinoma mouse models, CXCR4 blockade promotes T cell tumor infiltration and is synergistic with anti-PD-1 therapy. It was a phase IIa, open-label, two-cohort study to evaluate the safety, efficacy and immunobiologic effects of the CXCR4 antagonist BL-8040 (motixafortide) with pembrolizumab and chemotherapy in metastatic pancreatic cancer. The primary endpoint was objective response rate. 22 patients received triple combination of motixafortide, pembrolizumab with chemotherapy, with an overall response rate of 32% and median duration of response of 7.8 months [41]. These results need further evaluation in larger randomized clinical trial.

## 8. Ongoing Trials

So far, the results of clinical trials of ICIs have shown no significant benefit of ICIs in advanced or metastatic pancreatic cancer. Efforts are needed to improve the immunogenicity of pancreatic cancer cells [42]. However, there are several ongoing clinical trials of ICIs alone or in combination with other targeted therapies or cytotoxic chemotherapy that will provide further guidance in terms of role of ICIs in management of pancreatic cancer. Additionally, there are several ongoing clinical trials studying the combination of immunotherapy with vaccines in an effort to improve effector T-cell response in pancreatic cancer [35]. Nivolumab plus dendritic cell vaccine combination has shown partial response in two out of six patients with overall survival of 13 months and 5 months, respectively [43]. Additionally, Soares et al. showed that the combination treatment of granulocyte macrophage colony-stimulating factor secreting PDA vaccine (GVAX) and PD-1 inhibitor significantly upregulated PD-L1 expression and improved survival compared with GVAX monotherapy or PD-1 inhibitor monotherapy [44]. Further studies are required in order to understand how the microenvironment of pancreatic adenocarcinoma can become immunogenic in order to provide better response to immunotherapy.

## 9. Hepatocellular Carcinoma

Hepatocellular carcinoma (HCC) is the most common primary liver cancer and the third most common cause of cancer mortality in the world with over 780,000 deaths occurred in 2018 [45,46]. Patients are usually diagnosed in an advanced stage and are not candidates for curative intent surgery or liver transplant. Most of the patients with liver limited disease are treated initially with loco–regional therapies including trans-arterial chemo-embolization, Y-90 radio-embolization, external beam radiation or other local modalities. Systemic treatment is routinely utilized if patient has metastatic disease or if local therapy is not feasible. There has been tremendous advancement in the systemic treatment of HCC in the recent past, with several approvals of targeted therapies and immunotherapies. There are several options available for HCC in advanced or metastatic setting including sorafenib, lenvatinib, atezolizumab/bevacizumab, regorafenib, cabozantinib, ramucirumab, nivolumab and pembrolizumab [47]. The most recent approval was of atezolizumab/bevacizumab in first line setting. There is a definite need to understand how to sequence these therapies in appropriate patient populations. Some of the ongoing trials may help understand some of the nuances in sequencing of these agents.

## 10. Role of ICIs in First Line Setting

For several years sorafenib was the standard of care in first line setting. Later on, lenvatinib was approved in the first line setting. Lenvatinib showed no non-inferiority to sorafenib however these agents had modest activity in advanced HCC with very low response rates and limited overall survival. Fortunately, most recently the combination of atezolizumab and bevacizumab was shown to provide a survival benefit. The IMbrave150 was an open label phase III clinical trial comparing the combination of atezolizumab and bevacizumab to sorafenib in patients with unresectable HCC who had not previously received systemic treatment [48]. Patients were randomized to receive either atezolizumab and bevacizumab or sorafenib until progression or toxicity. At 12 months, OS was 67.2% (95% confidence interval, 61.3 to 73.1) with atezolizumab and bevacizumab and 54.6% (95% CI, 45.2 to 64.0) with sorafenib. The median PFS was 6.8 months (95% CI, 5.7 to 8.3) for atezolizumab and bevacizumab and 4.3 months (95% CI, 4.0 to 5.6) in the sorafenib group [29]. Due to the impressive results from this trial, the FDA granted approval to combination therapy of atezolizumab with bevacizumab in first line setting.

There have been several other trials that have looked into the role of immunotherapy in first line setting. Check-Mate 459 was a phase III randomized clinical trial comparing first line nivolumab to sorafenib in systemic therapy naive adults with advanced HCC [49]. Patients were randomized to nivolumab or sorafenib. OS did not meet the predefined threshold statistical significance (HR = 0.84, *P* = 0.0419), but median OS was 16.4 months for nivolumab and 14.7 months for sorafenib (HR = 0.85, *P* = 0.0752) and ORR was 15% for nivolumab and 7%. Nivolumab has not been approved in first line setting.

## 11. Role of ICIs in Second Line Setting and Beyond

In 2017, the FDA granted approval for nivolumab as a second line treatment in advanced HCC patients based on the phase I/II Check-Mate 040 study. This was an open label, noncomparative dose escalation and expansion trial of nivolumab in adults with histologically confirmed advanced HCC [50]. Forty-eight patients were in the dose-escalation phase and 214 in the dose-expansion phase. In patients treated with nivolumab 3 mg/kg, ORR was 20% for patients in the dose expansion phase and 15% in the dose escalation phase. The disease control rates were 64% and 58% for these phases. Nine-month OS for patients in the dose expansion phase was 74%. Eighteen-month OS rates for these patients were 57% and 44%, respectively. These promising results lead to Check-Mate 459, which was previously discussed.

The combination of nivolumab with ipilimumab has also been approved in second line setting for advanced HCC. The approval was part of CheckMate 040 trial, in which there was a randomized multicohort phase II study assessing the combination of nivolumab and ipilimumab in patients previously treated with sorafenib [51]. Patients were randomized to nivolumab 1 mg/kg plus ipilimumab 3 mg/kg every 3 weeks for four doses, followed by nivolumab 240 mg every 2 weeks, nivolumab 3 mg/kg plus ipilimumab 1 mg/kg every three weeks, for four doses, followed by nivolumab 240 mg every two weeks or nivolumab 3 mg/kg every two weeks plus ipilimumab 1 mg/kg every six weeks. ORR was 31% in combination arm, twice as high as the 14% seen with nivolumab alone. However, 37% of patients had a grade 3–4 immune mediated adverse events with combined therapy. Based on this study, the combination of nivolumab and ipilimumab was granted accelerated approval for treatment of HCC in patients previously treated with sorafenib. The recommended dose of nivolumab is 1 mg/kg followed by ipilimumab 3 mg/kg on the same day every 3 weeks for 4 doses, followed by nivolumab 240 mg every 2 weeks or 480 mg every 4 weeks.

In 2018, the FDA granted accelerated approval of pembrolizumab for second line treatment in advanced HCC based on KEYNOTE-224 trial. This was a non-randomized, multicenter, open label, phase II trial [52]. The patients involved in the trial had pathologically confirmed HCC and had previously been on sorafenib. This trial showed 17% ORR with pembrolizumab and 44% had stable disease. In another trial KEYNOTE-240, which was designed to confirm findings from the KEYNOTE-224 trial that led to the FDA’s accelerated approval for the pembrolizumab. This was a randomized, double-blind, phase III study that examined patients with advanced HCC, previously treated with sorafenib, who received pembrolizumab and best supportive care or placebo and best supportive care [53]. The predefined primary endpoints of OS and PFS (*P* = 0.0174 and *P* = 0.002, respectively) were not met, but median OS was 13.9 months for pembrolizumab and 10.6 months for placebo (HR = 0.781, *P* = 0.0238) and median PFS was 3 months for pembrolizumab and 2.8 months for placebo (HR = 0.718, *P* = 0.0022). However, safety and efficacy data were similar to KEYNOTE-224.

## 12. Ongoing Trials

There are currently ongoing ICI combination studies including ICIs with TGF-beta inhibitors, with indoleamine dioxygenase inhibitors, with intra-arterial therapies, with radiation and with angiogenesis inhibitors. Some ongoing Phase III ICIs combination studies include: Nivolumab plus ipilimumab in advanced HCC as first line therapy (NCT03510871, NCT03222076, NCT03203304, NCT01658878, NCT04039607) and durvalumab plus tremelimumab in advanced HCC as second line therapy (NCT03298451) [54]. Phase III studies involving ICI and angiogenesis inhibitors include: nivolumab plus sorafenib in advanced HCC as first line therapy (NCT02576509, NCT01658878, NCT03439891), pembrolizumab plus lenvatinib in patients with advanced HCC as first line therapy (NCT03713593), atezolizumab plus cabozantinib in advanced HCC as first line therapy (NCT03755791), atezolizumab plus bevacizumab in advanced HCC as first line therapy (NCT03434379), durvalumab plus bevacizumab in localized and locally advanced HCC (NCT03847428, NCT03778957), camrelizumab plus apatinib in advanced HCC as first line therapy (NCT02942329, NCT03764293), tislelizumab plus sorafenib for advanced HCC as first line therapy (NCT03412773) and sintilimab plus IBI305 in advanced HCC as first line therapy (NCT03794440) [54]. All studies involving ICIs plus locoregional therapies (radiofrequency ablation, radiotherapy or intra-arterial treatments), ICIs plus chemotherapy, ICIs plus TGF-beta inhibitors, ICIs plus indoleamine dioxygenase inhibitors and others are all in phase I/II stages [54]. Hopefully, the results of these trials will further help enhance the impact of immunotherapy in management of HCC.

## 13. Biliary Tract Cancers

Biliary tract tumors are rare tumors that include intrahepatic cholangiocarcinoma, extrahepatic cholangiocarcinoma and gallbladder carcinoma. They account for approximately 3 percent of all gastrointestinal cancers [55]. The outcome is dismal if the cancer is diagnosed in advanced or metastatic stage with 5 year survival of 10% [56]. Similar to HCC, they are usually diagnosed in an advanced stage and not candidates for curative intent surgery. There are several cytotoxic chemotherapies available for advanced, metastatic biliary cancers including gemcitabine with cisplatin and fluoropyrimidine based combinations. In second line setting targeted therapies against IDH and FGFR can be considered as well. However, there is no immunotherapy or ICI approved in biliary cancers. Similar to other tumors, ICIs can be given if the tumor has MSI-H or high TMB, but other than that ICIs are not approved by FDA in advanced or metastatic biliary cancers. There are several ongoing clinical trials that are exploring the role of immunotherapy in this rare tumor type.

There is currently no FDA approved first line immunotherapy for biliary tract cancers. Standard first line treatment for advanced biliary tract cancers involves gemcitabine based regimens usually combined with a platinum agent based on the ABC-02 trial [57]. Unfortunately, there is a lack of phase III data currently available, but there are several phase III ICI trials currently underway.

There is currently no established standardized second line treatment for biliary tract cancers. Multiple small phase I and II studies have assessed ICIs in biliary tract cancers after patients have failed at least one standard treatment regimen or have had inability to receive standard treatment.

There have been several studies using pembrolizumab as monotherapy in biliary tract malignancies. KEYNOTE 028 was a phase IB study using pembrolizumab monotherapy in pretreated patients with PD-L1 positive biliary tract cancer [58]. Results demonstrated an ORR of 13% among 24 patients with biliary tract cancers treated with pembrolizumab. KEYNOTE-158, was a nonrandomized, open label, multisite phase II study that enrolled patients with histologically or cytologically confirmed MSI-H/dMMR advanced non-colorectal cancer, including biliary adenocarcinoma, who experienced failure with prior therapy received pembrolizumab 200 mg once every 3 weeks for 2 years until disease progression, unacceptable toxicity or patient withdrawal. Results from this trial demonstrated ORR of 5.8%.

## 14. Ongoing Trials

Further studies using ICI monotherapy and ICIs in combination with other therapies are ongoing. There are ongoing phase II trials with first line combination nivolumab plus ipilimumab in advanced cholangiocarcinoma (NCT03101566, NCT02834013), second line pembrolizumab monotherapy in advanced cholangiocarcinoma (NCT03110328, NCT02628067), second line nivolumab in advanced cholangiocarcinoma (NCT02829918), first line durvalumab and tremelimumab with chemotherapy in advanced cholangiocarcinoma (NCT03473574, NCT03046862, NCT03704480), first line toripalimab with chemotherapy in advanced cholangiocarcinoma (NCT03796429, NCT03982680, NCT04027764) [54]. There are ongoing phase III trials of first line durvalumab with chemotherapy for advanced cholangiocarcinoma (NCT03875235) and first line pembrolizumab with chemotherapy in advanced cholangiocarcinoma (NCT03260712, NCT03111732, NCT04003636) [54]. There is a huge unmet need to have efficacious drugs for this tumor type in advanced setting, considering the overall survival is dismal in this disease. Hopefully, the future studies may help improve the outcomes of these patient and prolong the survival.

## 15. Colorectal Cancer

Colorectal cancer is the 2nd leading cause of cancer death globally, accounting for approximately 880,000 deaths in 2018 [45]. It is the second most common cause of cancer death in the United States when rates of men and women are combined, with approximately 147,950 cases diagnosed this year in the United States [59]. While surgical resection offers a curative option for localized colon cancer, about 20%–25% of newly diagnosed colon cancers are metastatic at presentation [60]. While a subset of the metastatic population with liver or lung isolated metastases or limited abdominal involvement still have chance for potentially curable surgery, for other patients with metastatic disease, the goal of systemic therapy is currently palliative. Although advances in chemotherapy have improved median survival rates overall, the five-year survival with metastatic colorectal cancer is still less than 20 percent of those treated with chemotherapy alone [61,62,63].

In addition to systemic chemotherapy, there have been promising immunotherapy options for subsets of colorectal cancer based on molecular markers. Currently, immune checkpoint inhibitors targeting PD-L1 and CTLA-4, are approved for first and second line therapy for metastatic colorectal cancer, specifically in patients with MSI-H/dMMR. Approximately 3.5% to 6.5% of all stage IV colorectal cancers have deficiency in mismatch repair enzymes [64]. Given the promising results in dMMR/MSI-H tumors, there are currently ongoing clinical trials for evaluation of various checkpoint inhibitors in first line, adjuvant and neoadjuvant settings [65]. Unfortunately, there are no immunotherapy agents approved for MSS colorectal cancer patients (except for patients with high TMB (≥10)). There are several ongoing combination trials to explore the role of immunotherapy in MSS colorectal cancer patients.

## 16. Role of ICIs in First Line Setting

First line therapy for unresectable metastatic colorectal cancer who are candidates for intensive regimens historically has been combination oxaliplatin based therapy (FOLFOX, CAPEOX, FOLFIRI, FOLFOXIRI) with or without an anti-VEGF agent, such as bevacizumab. Anti-epidermal growth factor (EGFR) monoclonal antibodies can be given with systemic chemotherapy for RAS/BRAF wild type tumors. Recently, immune checkpoint inhibitors have been approved for first line treatment for colorectal cancer in MSI-H tumors based on results from the pivotal KEYNOTE-177 trial. KEYNOTE-177 was a randomized, open label phase III study that compared pembrolizumab with standard of care chemotherapy as first line treatment for metastatic dMMR/MSI-H colorectal cancer [66]. In this trial patients were randomized to either pembrolizumab or investigators’ choice of standard of care chemotherapy. The co-primary endpoints were overall survival (OS) and progression free survival (PFS). 307 previously untreated patients with MSI-H/dMMR metastatic colorectal cancer were randomized to pembrolizumab at 200 mg every 3 weeks for up to 2 years or investigator’s choice of modified FOLFOX6 or FOLFIRI every 2 weeks, with or without bevacizumab or cetuximab. Treatment was continued until progressive disease or unacceptable side effects. Crossover was allowed in this trial. ORR were 43.8% and 33.1% in pembrolizumab and chemotherapy arms, respectively. Median PFS was 16.5 months in the Pembrolizumab group and 8.2 months in the chemotherapy group. Based on these results, the FDA granted approval to Pembrolizumab for first line treatment in dMMR/MSI-H colorectal cancer. This is the first immunotherapy for this patient population approved for first line treatment without concomitant chemotherapy.

There are a few other clinical trials that have investigated potential use of checkpoint inhibitors in previously untreated metastatic colorectal cancer. Part of the phase II trial Check-Mate 142 evaluated the efficacy and safety nivolumab and low dose ipilimumab in dMMR/MSI-H metastatic colorectal cancer patients who were not previously treated [67]. The primary endpoint was ORR and patients were treated until disease progression. A total of 45 patients were enrolled. The ORR was 60%, disease control rate was 84% and 7% of patients had complete response. The 12-month PFS was 77% and overall survival rate was 83%. There are currently other clinical trials underway evaluating the role of anti-PD-L1 inhibitors in first line therapy. One such trial for patients with dMMR metastatic colorectal cancer is the ongoing phase III COMMIT trial (NCT02997228) in which patients were randomized into one of 3 arms: standard chemotherapy + bevacizumab with atezolizumab, standard chemotherapy + bevacizumab without atezolizumab or atezolizumab monotherapy. Investigators’ primary endpoint is PFS with secondary endpoints of OS and objective response rate [68].

## 17. Role of ICIs in Second Line Setting

For patients who progress after first line therapy, the current therapeutic options include an irinotecan including regimen if they were previously treated with FOLFOX or XELOX and oxaliplatin based regimen if they previously were treated with FOLFIRI. Similar to first line therapy, bevacizumab/cetuximab can be added to RAS wild type tumors. Another option is encorafenib along with cetuximab for BRAF mutation positive tumors. For dMMR/MSI-H tumors that have progressed, the combination of nivolumab and ipilimumab or pembrolizumab are currently approved for second line treatment. However, there is no approved agent for MSS disease in second line setting.

The clinical activity of pembrolizumab was studied in 41 patients with progressive metastatic colorectal carcinomas, with and without mismatch-repair deficiency [69]. Primary endpoints included immune-related objective response rate and immune-related PFS. The objective response rate was 40% and PFS for mismatch-repair deficiency tumors was 78% compared to 0% response rate and 11% progression free survival for mismatch proficient tumors. In a follow-up study with a larger cohort, patients with dMMR metastatic colorectal cancer had a 50% objective response rate [70]. Based on this data, the FDA approved pembrolizumab for advanced MSI-H/dMMR colorectal cancer that had progressed after systemic chemotherapy. In KEYNOTE-164, a multicenter open-label study, Pembrolizumab showed a 33% percent objective response rate and was determined that it could be safely used in patients with refractory metastatic MSI-H/dMMR colorectal cancers [71].

The combination of nivolumab and ipilimumab is currently being studied in patients with metastatic MSI-H/dMMR colorectal cancer in a phase II open label multicenter study, CheckMate 142 [72]. The trial includes data from 31 sites globally and eligible patients are those who have progressed or have been intolerant of at least one previous line of treatment, including a fluoropyrimidine and oxaliplatin or irinotecan. The purpose is to assess the safety and activity of nivolumab with and without ipilimumab. In one analysis, out of 74 patients with dMMR metastatic colorectal cancer treated with nivolumab alone, 23 patients had an objective response (31%). The FDA approved nivolumab in August 2017 for MSI-H/dMMR metastatic colorectal cancer that had progressed following initial treatment. A later report showed that the combination with ipilimumab had greater benefit than nivolumab alone, in a nonrandomized comparison. There are currently no randomized clinical trials comparing dual immunotherapy compared to monotherapy; however indirect comparisons from Check-Mate 142 have suggested that combined therapy has improved efficacy. Large randomized clinical trials are still needed in order to show the relative risks and benefits of combined immunotherapy compared to monotherapy.

## 18. Role of ICIs in Third Line Setting

For tumors that are refractory to all standard combination chemotherapy options, the multiple protein kinase inhibitor regorafenib is approved as monotherapy for patients not candidates for any other available therapies. Another option for refractory tumors is trifluridine/tipiracil. There are several ongoing studies combining checkpoint inhibitors and different agents, including kinase inhibitors such as regorafenib, standard chemotherapy, anti-VEGF antibodies, and MEK inhibitors. MEK inhibition (cobimetinib) combined with anti-PD-L1 agents was hypothesized to help the activity of PD-L1 inhibition. These trials have been done in refractory metastatic colorectal cancer, not only on dMMR/MSI-H tumors.

The IMBlaze370 trial illustrated that there was no overall survival benefit with atezolizumab with or without cobimetinib when compared to regorafinib for patients who had progressed past 2 previous systemic chemotherapy regimens [73]. 5% of the patients in this study had high MSI. This study illustrated the challenges of extending therapy to non-MSI-H tumors. Another Phase I clinical trial is evaluating the safety of regorafenib and nivolumab in refractory pMMR/MSI-L colorectal cancers with a secondary endpoint of overall survival [74]. Another ongoing trial is a Phase I study (NCT03657641) evaluating the combination of regorafenib and pembrolizumab in metastatic colorectal cancer that has progressed beyond standard therapy [75]. Combinations of immune checkpoint inhibitors with anti-VEGF blockade is also being studied. One trial evaluated atezolizumab and bevacizumab with and without chemotherapy in refractory pMMR/MSI-L colorectal cancer [76].

## 19. Ongoing Trials

Currently immune checkpoint inhibitors are approved in the second line setting for dMMR/MSI-H tumors. However, many ongoing studies are currently evaluating the role of immunotherapy as first line treatment in these tumors. In addition, the role of adjuvant/neoadjuvant therapy with these inhibitors is still being investigated. One example is the phase II study (NCT02375672) that combines pembrolizumab with chemotherapy compared to pembrolizumab alone [77].

dMMR/MSI-H tumors have a high mutation burden and are heavily infiltrated by tumor infiltrating lymphocytes which make them more susceptible to T-cell activation and cytotoxic killing of tumor cells. However, pMMR/MSI-L colorectal cancers which represent most metastatic tumors have still not been successfully targeted with immune checkpoint inhibitors. In studies such as Check-Mate 142, limited response was seen in pMMR tumors. Similarly, the IMBlaze study also emphasized the lack of responsiveness in these tumors. Combination trials as described above have been using different approaches to create a more susceptible environment for checkpoint inhibitors in pMMR tumors. Entinostat, a class I-selective histone deacetylase inhibitor [78] has been shown to enhance anti-PD1 activity by downregulation of immunosuppressive cell types in vivo [79]. Entinostat has shown potential in lung cancer and melanoma. Based on these studies, there is an ongoing phase II study, ENCORE 601 (NCT02437136), that evaluated the safety and efficacy of Entinostat along with Pembrolizumab in MSS/pMMR colorectal cancer patients. Preliminary results are promising and show acceptable safety so far [80]. Other combinations include radiotherapy, which causes DNA damage and potentially can improve pembrolizumab response. Dual immune checkpoint inhibition with anti-CTLA4 and anti-PD-L1 agents combined with radiofrequency ablation is also being studied. Other approaches include vaccines to activate the tumor associated macrophages and aid PD-1 targeted therapies. However, the phase 2 study investigated the GVAX colon vaccine and pembrolizumab in pMMR colorectal cancer failed to meet its primary objective of ORR [81].

Immune checkpoint inhibitors have opened the door to a wide variety of therapeutic options for colorectal cancer in various settings. There are still challenges such as predicting response and resistance in dMMR/MSI-H tumors and also broadening therapeutics to target pMMR/MSI-L colorectal cancers. The results of ongoing trials will shape the future of colorectal cancer treatment and will give insight into the mechanisms of tumor environment in its response to immunotherapy.

## 20. Anal Squamous Cell Cancer

The incidence of anal squamous cell cancer has been increasing over the last several years. Most of the anal canal cancers are squamous cell carcinomas. The standard treatment of localized disease is usually combination of concurrent chemotherapy with radiation therapy. The chemotherapy regimen of choice in locoregional disease requires mitomycin with addition of 5-fluorouracil or capecitabine and concurrent radiation therapy [82]. Patients usually respond very well to concurrent chemotherapy and radiation therapy with long-term survival [83]. However, twenty percent of patients with anal SCC present with extra-pelvic metastases [84], commonly to the liver and lung. In metastatic disease, chemotherapy with or without radiation therapy is standard of care. There are several cytotoxic chemotherapies that are utilized in treatment of advanced or metastatic anal squamous cell carcinoma. The phase II InterAACT study outlined that carboplatin and paclitaxel is the preferred regimen due to fewer side effects compared to other regimens [85]. However, there are other approved chemotherapy agents that are as effective which include cisplatin and infusional 5-fluorouracil; modified 5FU, leucovorin, oxaliplatin (mFOLFOX); 5-FU, leucovorin, cisplatin; or docetaxel plus cisplatin with infusional 5-FU [85]. There is approved immunotherapy agent for treatment of anal squamous cell carcinoma. However, ICIs can be considered in chemotherapy resistant tumors.

Immune checkpoint inhibitors have shown benefit in patients with refractory metastatic anal SCC after chemotherapy [86]. Several checkpoint inhibitors, including nivolumab, pembrolizumab and durvalumab have been studied in the metastatic setting. The NCI9673 study was a multicenter phase II trial where 37 patients were able to receive at least one dose of nivolumab at three milligrams per kilogram intravenously every two weeks [87]. The median number of cycles given was six with two patients with complete response and 46% of patients with stable disease. Median PFS was 4.1 months with a median OS of 11.5 months. PD-L1 expression was not required to be measured however there was a correlation to response in patients that showed overexpression of PD-L1. The side effect profile was comparable to its known side effects including pneumonitis (grade 2) and anemia (grade 3) in about 5% of the patients. In about 3% of patients grade 3 fatigue, rash and hypothyroidism was observed, but overall nivolumab was well tolerated.

Pembrolizumab has also been studied in anal SCC. It was initially studied in the KEYNOTE-028 trial, a phase Ib multicohort trial where 24 PD-L1 positive patients were found to have an overall response rate of 17% with 42% having stable disease [88]. Among the 24 patients with anal SCC histology, four patients had confirmed partial responses with ORR of 17%, with 10 other patients having stable disease (42%). The median PFS was 3.0 months and median overall survival was 9.3 months which were similar between the previously reported nivolumab studies. The most common treatment-related adverse events were diarrhea, fatigue and nausea. Pembrolizumab and nivolumab were well tolerated in the second line refractory metastatic setting and are accepted as an immunotherapeutic option per the NCCN guidelines.

It is important to mention that there are associated viruses that are related to anal squamous cell carcinoma which include HPV and HIV. HPV is seen in at least 90% of patients that are diagnosed with anal squamous cell carcinoma. HPV 16 and 18 are the most common type of HPV seen in anal SCC [84]. The rate of patients with HIV developing anal SCC has increased over the years, about 78 per 100,000 person-years [89]. Generally many of the trials listed above included patients with HIV, but required CD4 counts greater than 300 and an undetectable viral load while being on antiretroviral therapy with close follow-up [90]. There have not been enough studies to understand if immunotherapy is completed contraindicated in these patients. However, a systematic review has suggested that immune checkpoint inhibitor therapy in patients with HIV infection was associated with no new safety concerns [91].

## 21. Ongoing Trials

The future of immunotherapy as a therapeutic option in anal SCC is an expanding field with many new trials looking at the utility of immune checkpoint inhibitors. There are many studies looking at a multi-targeted approach as well as combinations with chemotherapy, radiation therapy and other immunotherapeutic agents at different stages of the disease. Currently, nivolumab monotherapy is being studied in the locally advanced setting (stages II and III) in a phase II trial [92]. Along with this there is a phase Ib/II trial (NCT04046133) looking at the use of pembrolizumab with IMRT in stage III anal SCC. Another trial is evaluating pembrolizumab in locally advanced and metastatic anal SCC patients who are unresectable [93].

Further in the realm of chemo–immunotherapy and radiotherapy, patients with early stage II and III anal SCC are being assessed with treatment of durvalumab along with standard of care mitomycin and 5-FU (NCT04230759) [94]. Similarly, modified DCF in combination with atezolizumab is being evaluated in the SCARCE trial (NCT03519295) [95]. Another study is evaluating the combination of avelumab combined with cetuximab (NCT03944252). This trial was based on a previous trial which did not meet its endpoint, but surprisingly it did allow for local control of the disease [96]. In addition, clinical trial (NCT02314169)looking at combination of nivolumab plus ipilimumab and trial NCT04444921 is looking at adding nivolumab to chemotherapy for metastatic anal squamous cell carcinoma is also ongoing [97]. More recently HPV related E6 and E7 proteins are being targeted. Trials assessing the role of CAR-T cell therapy [98,99] and a listeria based immune vaccine in combination with chemo–radiotherapy are underway [100].

## 22. Conclusions

Immune checkpoint inhibitors have been demonstrated to be effective in many gastrointestinal malignancies. They have provided new avenue for management for advanced gastrointestinal malignancies which are historically treated with cytotoxic chemotherapies. Immune checkpoint inhibitors are specifically approved in treatment for gastroesophageal cancers and hepatocellular carcinoma but can also be utilized in MSI-H/dMMR tumors of gastrointestinal tract. In addition to that patient with high tumor mutational burden can also be considered for immune checkpoint inhibitors in refractory settings. It is imperative to routinely check the status of certain biomarkers for immunotherapy including MSI, tumor mutational burden and PDL-1 to determine the role of immunotherapy. There is still a huge unmet need to expand their use and improve their efficacy. The challenge is determining which patients will benefit from these treatments. Future studies are evaluating not only monotherapy with immune checkpoint inhibitors, but also combinations of these inhibitors with chemotherapy, immune-modulatory agents, radiation and vaccines in order to potentiate the effect of immunotherapy across various gastrointestinal malignancies. Further research needs to be done to determine valid prognostic and predictive biomarkers that can identify suitable patients for this type of therapy. As data from ongoing trials emerges, immune checkpoint inhibitors may become standard of care in earlier lines of therapy for various gastrointestinal malignancies and hopefully help improve overall outcomes in foreseeable future. (Table 2 and Table 3)

## Figures and Tables

**Table 1:** Relevant Trials for FDA Approved Immunotherapy.

Line of Therapy	Name of Drug	Trial Name	Trial Design	Standard Arm	Objective Response Rate	Overall Survival	Progression Free Survival	PDL1 Status
**Squamous Cell Esophageal Cancer**								
Second	Pembrolizumab	Keynote 181	open label randomized phase III	chemotherapy (paclitaxel, docetaxel, irinotecan)	22% vs 7% (chemo)	10.3 months vs 6.7 months (chemo)	3.2 months vs 2.3 months (chemo)	CPS >10
Second	Pembrolizumab	Keynote 180	open label single arm phase II	none	14.3% (SCC)	5.8 months	2 months	CPS >10
Second	Nivolumab	Attraction 3	open label randomized phase III	chemotherapy (paclitaxel or docetaxel)	19.3% vs 21.5% (chemo)	10.5 months vs 8.4 months (chemo)	1.7 months vs 3.4 months (chemo)	no
**Gastroesophageal/Gastric Cancer**								
Third	Pembrolizumab	Keynote 059	open label single arm phase II	none	11.6%	5.6 months	2 months	CPS>1
**Hepatocellular Cancer**								
Second	Pembrolizumab	Keynote 244	open label, non-randomized Phase II	none	17%	12.9 months	4.9 months	no
Second	Nivolumab	Checkmate 040	open label, non-comparative, dose escalation and expansion Phase I/II	none	15% in dose escalation phase; 20% in dose expansion phase	83% at 6 months; 74% at 9 months	37% at 6 months; 28% at 9 months	no
Second	Nivolumab + Ipilimumab	Checkmate040	open label, non-comparative, dose escalation and expansion Phase I/II	none	33%	23 months	88% at 6 months; 31% at least 24 months	no
First*	Atezolizumab + Bevacizumab	IMbrave 150	open label randomized Phase III	sorafenib	28% vs 12% (sorafenib)	Not est. vs 13.2 months (sorafenib)	6.8 months vs 4.5 (sorafenib)	no
**Colorectal Cancer**								
First for dMMR/MSI-H	Pembrolizumab	Keynote 177	Randomized Phase III	chemotherapy (mFOLFOX6/ FOLFIRI +/- bevacizumab or cetuximab	43.8% vs 33.1% (chemo)	Not achieved vs 10.6 months (chemo)	16.5 months vs 8.2 months (chemo)	no
Second for dMMR/MSI-H	Pembrolizumab	Keynote 164	open label single arm Phase II	none	33%	31.4 months	2.3 months	no
Second for dMMR/MSI-H	Nivolumab	Checkmate 142	open label single arm Phase II	none	31%	73% at 12 months	50.4% at 12 months	no
Second for dMMR/MSI-H	Nivolumab + Ipilimumab	Checkmate 142	open label single arm Phase II	none	46%	85% at 12 months	71% at 12 months	no

**Table 2:** Completed phase III trials of immunotherapy in advanced gastrointestinal cancers.

NCT Number	Line of Therapy	Name of Drug	Trial Name	Standard Arm	Objective Response Rate	Overall Survival	Progression Free Survival	PDL1 Status
Gastroesophageal Cancer								
NCT 02564263	Second	Pembrolizumab	Keynote 181	Chemotherapy (paclitaxel, irinotecan or docetaxel)	22% vs 7% (chemo)	10.3 months vs 6.7 months (chemo)	3.2 months vs 2.3 months (chemo)	CPS> 10
NCT 02569242	Second	Nivolumab	Attraction 3	Chemotherapy (docetaxel or paclitaxel)	19% vs 22% (chemo)	10.9 months vs 8.4 months	not significant	no
NCT 02625623*	Second	Avelumab	JAVELIN Gastric 300	chemotherapy (paclitaxel or irinotecan)	2.2% vs 4.3% (chemo)	4.6 months vs 5 months (chemo)	1.4 months vs 2.7 months (chemo)	no
NCT 02370498	Second	Pembrolizumab	Keynote 061	chemotherapy (paclitaxel)	Not reached	9.1 months vs 8.3 months (chemo)	1.5 months vs 4.1 months (chemo)	no
NCT 02494583	First	Pembrolizumab	Keynote 062	chemotherapy (cisplatin + 5FU)	57.1% vs 36.8% (chemo)	10.6 months vs 11.1 months (chemo)	2 months vs 6.4 months (chemo)	CPS>1 and CPS >10
NCT 02494583	First	Pembrolizumab + chemotherapy	Keynote 062	chemotherapy (cisplatin + 5FU)	64.7% vs 36.8%	12.5 months vs 11.1 months (chemo)	6.9 months vs 6.4 months (chemo)	CPS >1 and CPS >10
NCT 02625610	First (after 12 weeks chemo)	Avelumab + chemotherapy	JAVELIN Gastric 100	chemotherapy (oxaliplatin + 5-FU capecitabine)	Not reached	10.4 months vs 10.9 months (chemo)	not reached vs 5.9 months (chemo)	no
Hepatocellular Cancer								
NCT 03434379	First	Atezolizumab + Bevacizumab	IMBrave 150	sorafenib	27% vs 12% (chemo)	Not estimable vs 13.2 months (chemo)	6.8 months vs 4.5 months (chemo)	no
NCT 02576509	First	Nivolumab	Checkmate 459	sorafenib	15% vs 7% (chemo)	16.4 months vs 14.7 months (chemo)	3.7 months vs 3.8 months (chemo)	no
NCT 02702401	Second	Pembrolizumab + Supportive Care	Keynote 240	supportive care	16.9% vs 2.2% (supportive)	13.9 months vs 10.6 months (supportive)	3.0 months vs 2.8 months (supportive)	
Colorectal Cancer								
NCT 02788279*	Third	Atezolizumab + Cobimetinib combo and Atezolizumab monotherapy	IMBlaze 370	Regorafenib	Not established	8.87 months (combo) vs 7.10 months (mono) vs 8.51 months (regorafenib)	Not established	no
NCT 02563002	First (dMMR/MSI-H)	Pembrolizumab	Keynote 177	chemotherapy (mFOLFOX6/ FOLFIRI +/- bevacizumab or cetuximab	43.8% vs 33.1% (chemo)	Not achieved vs 10.6 months (chemo)	16.5 months vs 8.2 months (chemo)	no

**Table 3:** Ongoing early phase clinical trials of immunotherapy in gastrointestinal cancers.

NCT Number	Line of Therapy	Drug Name	Trial Name	Trial Design	Standard Arm	PDL1 Status
Gastro-Esophageal Cancer						
NCT 02954536	First	Pembrolizumab + Trastuzumab and chemotherapy	A Phase II Trial of Pembrolizumab With Trastuzumab and Chemotherapy in Advanced HER2+ Esophagogastric Cancer	phase II	none	no
NCT 02639065	First	Durvalumab	Study of Durvalumab (MEDI4736) in Esophageal Cancer	open label phase II	none	no
NCT 02559687	Third	Pembrolizumab	KEYNOTE-180	phase II	none	no
NCT 02689284	First/ Second	Pembrolizumab + Margetuximab	Combination Margetuximab and Pembrolizumab for Advanced, Metastatic HER2(+) Gastric or Gastroesophageal Junction Cancer	phase 1b/2 open label dose escalation study	none	no
NCT 02335411	First/ Second	Pembrolizumabor Pembrolizumab + chemotherapy	KEYNOTE-059	phase II	none	no
Pancreatic Cancer						
NCT 2826486	Second	Motixafortide + Pembrolizumab	COMBAT/KEYNOTE-202	randomized phase IIa	BL-8040	no
NCT 3184870	Second	BMS-813160 + Nivolumab	A Phase 1b/2 Study of BMS-813160 in Combination with Chemo or Nivolumab in Patients with Advanced Solid Tumors	non-randomized phase I/II	none	no
NCT 3193190	Second	Atezolizumab + chemotherapy + Selicrelumab	Morpheus-Pancreatic Cancer	randomized phase Ib and II	chemotherapy (nab-paclitaxel and gemcitabine)	no
NCT 03849469	Second	XmAb22841 + Pembrolizumab	DUET-4	nonrandomized phase I	none	no
NCT 03257761	Second	Guadecitabine, Durvalumab	A Phase Ib Study of Guadecitabine and Durvalumab in Patients with Advanced Hepatocellular Carcinoma, Pancreatic Adenocarcinoma, GB cancer, and Cholangiocarcinoma	phase Ib	none	no
NCT 04361162	Second (MSI stable)	Nivolumab + Ipilimumab	Nivolumab anD Ipilimumab and Radiation Therapy in Metastatic, Microsatellite Stable Pancreatic Cancer	phase II	none	no
NCT 03816358	Second	Anetumab Ravtansine, Nivolumab, Ipilimumab	A Phase I Study of Anetumab Ravtansine in Combination with Either Anti-PD-1 Antibody, or Anti-CTLA4 and Anti-PD-1 Antibodies or Anti-PD-1 Antibody and Gemcitabine in Mesothelin-Positive Advanced Pancreatic Adenocarcinoma	Non-randomized phase I/II	Anetumab ravtansine, nivolumab, gemcitabine	no
NCT 04161755	First	Atezolizumab	Phase 1 Clinical Trial of Personalized Neoantigen Tumor Vaccines and Programmed Death-Ligand 1 (PD-L1) Blockade in Patients with Surgically Resected Pancreatic Cancer	phase I	Chemotherapy (mFOLFIRINOX)	no
NCT 03563248	First	Losartan + Nivolumab	A Randomized Phase 2 Study of Losartan and Nivolumab in Combination With FOLFIRINOX and SBRT in Localized Pancreatic Cancer	randomized phase II	chemotherapy (FOLFIRINOX)	no
Hepatocellular Cancer						
NCT 04170556	Second	Regorafenib + Nivolumab	The GOING Study: Regorafenib Followed by Nivolumab in Patients With Hepatocellular Carcinoma Progressing Under Sorafenib	phase II	none	no
NCT 03316872	Second	Pembrolizumab + Radiotherapy	Pembrolizumab and Stereotactic Radiotherapy Combined in Subjects With Advanced Hepatocellular Carcinoma	phase II	none	no
NCT 04152356	First	Sorafenib + anti-PDI	Study on Combined Immunotherapy and Targeted Therapy for Hepatocellular Carcinoma	phase II	none	no
NCT 02821754	First	Durvalumab + Tremelimumab with or without TACE/RFA/cryo	A Pilot Study of Combined Immune Checkpoint Inhibition in Combination With Ablative Therapies in Subjects With Hepatocellular Carcinoma (HCC) or Biliary Tract Carcinomas (BTC)	phase II	none	no
NCT 03841201	First	Nivolumab + Lenvatinib	IMMUNIB trial	open label phase II	none	no
NCT03753659	First	Pembrolizumab + Ablation	IMMULAB - A Phase II Trial of Immunotherapy With Pembrolizumab in Combination With Local Ablation for Patients With Early Stage Hepatocellular Carcinoma (HCC)	phase II	none	no
Biliary Tract Cancer						
NCT 02866383	Second	Nivolumab + radiotherapy or Nivolumab/ Ipilimumab + radiotherapy	A Prospective Randomized, Open-label Phase 2 Study of Immune Checkpoint Inhibition, Nivolumab With or Without Ipilimumab in Combination With Radiation Therapy in Pretreated Patients With Metastatic Pancreatic Cancer or Biliary Tract Cancer.	randomized open label phase II	none	no
NCT 03110328	Second	Pembrolizumab	Phase II Study of Pembrolizumab in Metastatic Biliary Tract Cancer as Second-line Treatment After Failing to at Least One Cytotoxic Chemotherapy Regimen: Integration of Genomic Analysis to Identify Predictive Molecular Subtypes	phase II	none	no
NCT 02829918	Second	Nivolumab	A Phase II Investigator Sponsored Study of Nivolumab in Patients With Advanced Refractory Biliary Tract Cancers	phase II	none	no
NCT 03999658	Second	STI-3031, an anti-PD-L1 antibody	An Open-label, Multicenter, Global Phase 2 Basket Study to Investigate the Efficacy, Safety, Pharmacokinetics and Pharmacodynamics of STI-3031 in Patients With Selected Relapsed or Refractory Malignancies	open label phase II	none	no
NCT 03250273	Second	Entinostat + Nivolumab	A Phase 2 Clinical Trial of Entinostat in Combination With Nivolumab for Patients With Previously Treated Unresectable or Metastatic Cholangiocarcinoma and Pancreatic Adenocarcinoma	Phase II	none	no
NCT 03473574	First	Durvalumab + Tremelimumab + chemotherapy	IMMUCHEC trial	randomized phase II	none	no
NCT 03796429	First	Toripalimib + chemotherapy	A Single-arm, Single-center, Prospective Clinical Study of the Efficacy and Safety of Chemotherapy Combined With Toripalimab in Treatment of Advanced Biliary Tract Cancer	phase II	none	no
NCT 03201458	First	Atezolizumab + Cobimetinib or Atezolizumab	A Randomized Phase 2 Study of Atezolizumab in Combination With Cobimetinib Versus Atezolizumab Monotherapy in Participants With Unresectable Cholangiocarcinoma	randomized phase II	none	no
NCT 03101566	First	Nivolumab + chemotherapy or Nivolumab + Ipilimumab	A Randomized Phase II Study of Nivolumab in Combination With Gemcitabine/Cisplatin or Ipilimumab as First Line Therapy for Patients With Advanced Unresectable Biliary Tract Cancer	randomized phase II	none	no
Colorectal Cancer						
NCT 03228667	Second (MSI-H)	ALT-803 + anti-PD-1/PDL-1 antibody	QUILT-3.055 trial	phase IIb	none	no
NCT 02484404	Second	MEDI4736, anti-PDL-1 + Olaparib and/or Cediranib	Phase I/II Study of the Anti-Programmed Death Ligand-1 Antibody MEDI4736 in Combination With Olaparib and/or Cediranib for Advanced Solid Tumors and Advanced Colorectal Cancers	phase I/II	none	no
NCT 02754856	Second	Tremelimumab + Durvalumab	Pilot Study Assessing the Safety and Tolerability of the Neoadjuvant Use of Tremelimumab (Anti-CTLA-4) Plus Durvalumab (MEDI4736) (Anti-PD-L1) in the Treatment of Resectable Colorectal Cancer Liver Metastases	phase II	none	no
NCT 02982694	Second (MSI-H)	Atezolizumab + Bevacizumab	A Phase II Open-label Study with the Anti-PD-L1 Atezolizumab Monoclonal Antibody in Combination With Bevacizumab in Patients With Advanced Chemotherapy Resistant Colorectal Cancer and MSI-like Molecular Signature	open label phase II	none	no
NCT 04118933	Second (MSI-H)	JSOO1 Anti PDL-1 antibody	An Exploratory Study for PD-1 Antibody JS001 in Participants With Microsatellite Instability-high (MSI-H) Advanced or Recurrent Colorectal Cancer	phase II	none	no
NCT 03206073	Second	Pexa-Vec + Durvalumab or Durvalumab + Tremelimumab	A Phase I/II Study of Pexa-Vec Oncolytic Virus in Combination with Immune Checkpoint Inhibition in Refractory Colorectal Cancer	phase I/II	none	no
NCT 03186326	Second (MSI-H)	Avelumab	Multicenter Randomized Phase II Study Comparing the Effectiveness and Tolerance of Avelumab Versus Standard 2nd Line Treatment Chemotherapy in Patients with Colorectal Metastatic Cancer with Microsatellite Instability	randomized phase II	Chemotherapy (FOLFOX/FOLFIRI +/- anti-VEGF)	no
NCT 03376659	Second	Durvalumab + CV301 + Chemotherapy	A Phase I/II Trial of the PD-L1 Inhibitor, Durvalumab Plus CV301 in Combination with Maintenance Chemotherapy for Patients with Metastatic Colorectal or Pancreatic Adenocarcinoma	phase I/II	none	no
NCT 03608046	Second (MSS)	Avelumab + Cetuximab/Irinotecan	AVETUXIRI Trial	Phase IIa	none	no
NCT 03642067	First (MSS)	Nivolumab + Relatimab	Phase 2 Study Evaluating Response and Biomarkers in Patients With Microsatellite Stable (MSS) Advanced Colorectal Cancer Treated With Nivolumab in Combination With Relatlimab	open label phase II	none	no
NCT 02811497	First (MSS)	Azacitidine + Durvalumab	METADUR trial	open label phase II	none	no
NCT 03202758	First	Durvalumab + Tremelimumab + FOLOFOX	Phase Ib/II Trial Evaluating the Safety, Tolerability and Immunological Activity of Durvalumab (MEDI4736) (Anti-PD-L1) Plus Tremelimumab (Anti-CTLA-4) Combined with FOLFOX in Patients with Metastatic Colorectal Cancer	Phase Ib/II	none	no
Anal Cancer						
NCT 02314169	Second	Nivolumab	A Multi-Institutional Phase 2 Study of Nivolumab or Nivolumab in Combination With Ipilimumab in Refractory Metastatic Squamous Cell Carcinoma of the Anal Canal	phase II	none	no
NCT 03519295	Second	Atezolizumab + chemotherapy	SCARCE trial	randomized phase II	chemotherapy (docetaxel, cisplatin, 5-FU)	no
NCT 03944252	second	Avelumab or Cetuximab + Avelumab	CARACAS trial	randomized phase II	none	no
NCT 04230759	first	Durvalumab	Radio-chemotherapy +/- Durvalumab for Locally-advanced Anal Carcinoma	randomized phase II	chemotherapy (5-FU + Mitomycin C)	no
NCT 04046133	first/ second	Pembrolizumab + chemotherapy	Phase 1b/II Trial of Pembrolizumab Plus IMRT in Stage III/IV Carcinoma of Anus	phase Ib/II	none	no
NCT 03233711	first	Nivolumab	Nivolumab After Combined Modality Therapy in Treating Patients With High Risk Stage II-IIIB Anal Cancer	randomized phase II	clinical observation	no
